# Perception of patients in relation to the humanized care provided by nursing

**DOI:** 10.15649/cuidarte.4477

**Published:** 2025-06-02

**Authors:** Katerine Correa-Yantany, Ximena Osorio-Spuler, Luis Bustos-Medina, María Cecilia Toffoletto, Sara Barrios-Casas

**Affiliations:** 1 Regional Hospital Coyhaique, Coyhaique, Chile. E-mail: k.blanco01@ufromail.cl Regional Hospital Coyhaique Coyhaique Chile k.blanco01@ufromail.cl; 2 Nursing Department, Universidad de La Frontera, Temuco, Chile. E-mail: ximena.osorio@ufrontera.cl Universidad de La Frontera Temuco Chile ximena.osorio@ufrontera.cl; 3 Public Health Department-CIGES, Universidad de La Frontera, Temuco, Chile. E-mail: luis.bustos@ufrontera.cl Universidad de La Frontera Temuco Chile luis.bustos@ufrontera.cl; 4 School of Nursing, Universidad de las Américas, Santiago, Chile. E-mail: mtoffoletto@udla.cl Universidad de las Américas Santiago Chile mtoffoletto@udla.cl; 5 Nursing Department, Universidad de La Frontera, Temuco, Chile. E-mail: sara.barrios@ufrontera.cl Universidad de La Frontera Temuco Chile sara.barrios@ufrontera.cl

**Keywords:** Humanization of Assistance, Quality of Health Care, Nurses, Humanización de la Atención, Calidad de la Atención de Salud, Enfermeras, Humanização da Assistência, Qualidade da Assistência à Saúde, Enfermeiras

## Abstract

**Introduction:**

Humanization of care is a need demanded by users, and it is necessary to generate evidence that identifies both strengths and shortcomings in nurse-patient care.

**Objective:**

To determine the perception of adult patients regarding the humanized care provided by nursing professionals in a hospital in the Southern Zone of Chile.

**Materials and Methods:**

A cross-sectional and analytical study was conducted using a sociodemographic survey and the third version of the Perception of Behavior Related to Humanized Nursing Care (PCHE) instrument, which reports an internal consistency determined by Cronbach's alpha of 0.96. A non-probabilistic convenience sampling method was applied, and the ethical principles proposed by Ezekiel J. Emanuel were upheld.

**Results:**

Overall, most patients perceived that nurses always delivered humanized care. When comparing the mean scores of the PCHE dimensions with sociodemographic variables, significant differences were found in relation to sex and educational level: men with secondary education reported higher scores.

**Discussion:**

The perception of humanized care among hospitalized adult patients was more favorable than in a Mexican study but lower than the findings of a Chilean study conducted in a Hemato-Oncology Unit.

**Conclusion:**

As the lowest scores were observed in the openness to nurse-patient communication dimension, developing strategies to strengthen this bond is essential.

## Resumen

**Introducción:**

La humanización en la atención es una necesidad que demandan los usuarios y es necesario generar evidencia que permita identificar tanto fortalezas como falencias en la atención enfermera-paciente.

**Objetivo:**

Determinar la percepción de pacientes adultos en relación con el cuidado humanizado otorgado por el profesional de enfermería en un hospital de la Zona Austral de Chile.

**Materiales y métodos:**

Se realizó un estudio analítico de corte transversal, mediante una encuesta sociodemográfica y la aplicación de la tercera versión del instrumento Percepción de Conductas Humanizadas de Cuidado de Enfermería (PCHCE), que reporta una consistencia interna determinada por alfa de Cronbach de 0,96. Se utilizó un muestreo no probabilístico por conveniencia y se respetaron los principios éticos propuestos por Ezekiel J. Emanuel.

**Resultados:**

A nivel global, la mayoría de los pacientes percibió que las enfermeras siempre brindaron un cuidado humanizado. Al comparar los puntajes promedio de las dimensiones del instrumento con las variables sociodemográficas, se encontraron diferencias significativas en relación con el sexo y el nivel educativo: los hombres con educación secundaria presentaron puntajes más altos.

**Discusión:**

La percepción del cuidado humanizado en pacientes adultos hospitalizados fue más favorable que en un estudio realizado en México, pero menor que lo reportado en una investigación chilena en una Unidad de Hemato-Oncología.

**Conclusiones:**

Dado que la dimensión con menor puntuación fue la apertura a la comunicación enfermera–paciente, es esencial desarrollar estrategias que fortalezcan este vínculo.

## Resumo

**Introdução:**

A humanização no atendimento é uma necessidade demandada pelos usuários, sendo necessário gerar evidências para objetivar potencialidades e fragilidades no cuidado enfermeiro-paciente.

**Objetivo:**

Determinar a percepção de pacientes adultos sobre o atendimento humano prestado pelos profissionais de enfermagem em um hospital da Zona Sul do Chile.

**Materiais e métodos:**

Foi realizado um estudo transversal e analítico, com inquérito sociodemográfico e o Instrumento Percepção do Cuidado Humanizado de Enfermagem 3ª versão, que apresenta consistência interna determinada pelo alfa de Cronbach de 0,96, amostragem não probabilística, por conveniência e respeitando os princípios éticos de Ezekiel J. Emanuel.

**Resultados:**

Em nível global, ficou evidente que a percepção dos pacientes era de que os enfermeiros sempre forneciam cuidados humanos. Ao comparar a pontuação média das dimensões com as variáveis sociodemográficas, houve diferenças significativas por gênero: os homens valorizaram mais quando tinham ensino médio.

**Discussão:**

A percepção favorável do atendimento humanizado entre pacientes adultos hospitalizados foi maior em outro estudo mexicano e menor do que a relatada em um estudo chileno.

**Conclusões:**

Considerando que o menor escore foi: abertura à comunicação enfermeiro-paciente, é fundamental criar estratégias que ajudem a fortalecer esse vínculo.

## Introduction

The nursing profession has been recognized for organizing, planning, prioritizing, and demonstrating expertise in interventions focused on patient care, drawing on a scientific knowledge base and also an understanding of the individual. However, care within healthcare institutions takes place in an environment of tension and conflict —between the ought-to-be caring oriented towards empathy for others and, at the same time, the system's demands for effectiveness and efficiency^1^. 

Over the past few years, society and nursing professionals have evolved alongside technological advances. The emergence of new tools has opened up new possibilities for treatment and care, leading to cultural and societal shifts^2^. 

In the health sector, technology has given rise to the new concept of Information and Communication Technologies (ICT), which are used in various ways. Therefore, no service remains unaffected by the influence of ICT, including areas such as health planning, electronic medical records, and telecare, among others^3^. 

ICT has also contributed to the four functions that nursing has to perform: administration, teaching, research, and care. The care function is particularly relevant, as it involves managing an electronic medical record and utilizing other technological tools that facilitate the nurse's work, while also enabling remote care and monitoring^3^. 

As previously mentioned, technological development, along with demographic and epidemiological shifts, has changed the delivery of nursing care. This change has contributed to patients feeling unprotected by the healthcare system and dissatisfied with the care they receive. Therefore, technology has not only contributed to dehumanization but also directly impacted the relationship between patients and healthcare professionals, creating barriers and widening the gap between them^4^,^5^. 

However, other factors have also triggered the dehumanization of care, including^6^:

- Social factors, given the incidence of cultural diversity among human groups. Within the cultural competence framework and current social dynamics of clinical nursing practice, care remains a constant challenge, as it must consider and accommodate individual values and beliefs.

- Organizational and institutional factors derived from the lack of personalized care in healthcare facilities and a work structure oriented toward technical tasks.

- Educational factors, where training often emphasizes biomedical content and technical procedures, along with the trend of hyper-specialization among healthcare professionals.

- Care-related factors. During their shifts, nurses are often required to perform tasks beyond their professional role due to a shortage of professional personnel in health facilities and the high user demand. This problem means that nurses have to carry out several processes within limited timeframes, requiring significant concentration and precision, which negatively impacts the nurse-patient relationship^7^.

Dehumanization of care encompasses various aspects, which has led to a search for answers regarding humanized care. Accordingly, the general objective of this study is to assess the perceptions of patients hospitalized in the adult Intermediate Treatment Unit (ITU), Medicine Service, and Surgery Service regarding the humanized care nurses provided in a hospital located in the Southern Zone of Chile in 2023. 

## Materials and Methods

 A cross-sectional, analytical study^8^ was conducted in a hospital in the Southern Zone of Chile. The study population included early, middle, or late adulthood patients hospitalized in the Intermediate Treatment Unit (ITU), Medicine Service, or Surgery Service during June and July 2023. A non-probabilistic convenience sampling method was used, resulting in a sample of 61 patients who met the following inclusion criteria: aged between 18 and 84 years; hospitalized for 48 hours or more in the specified services during the study period; signed the informed consent form; had adequate cognitive and visual condition to complete the instrument and survey, and did not present any neurological impairments.

 For data collection, a sociodemographic survey was used consisting of 13 questions covering the following variables: sex, age, place of origin (urban or rural), type of health insurance, ethnicity, educational level, religion, marital status, support network, hospitalization service, role within the family group, living arrangement, and nationality. In addition, the third version of the Perception of Behavior Related to Humanized Nursing Care (PCHE, by its Spanish acronym) instrument was applied. This instrument includes 32 items across three dimensions, using a 4-point Likert-type scale (1 = never, 2 = sometimes, 3 = almost always, and 4 = always). The first dimension refers to nursing qualities, the second refers to openness to nurse-patient communication, and the third refers to the willingness to provide care^9^.

 The PCHE instrument demonstrated a content validity index of 0.98. Its reliability, assessed through internal consistency, yielded a Cronbach's alpha of 0.96^9^. Therefore, these results indicated that the instrument was reliable for use in this study.

**Data analysis **

 Data analysis was performed using Stata software, version 18. For the sociodemographic variables, mean, standard deviation, minimum and maximum values, and percentages were calculated. . For the third version of the PCHE instrument, frequencies and percentages were reported. Mean, standard deviation, minimum, and maximum values were also computed for its dimensions. To examine associations between PCHE scores and sociodemographic variables, t-tests for unequal variances, analysis of variance (ANOVA), and the Kruskal–Wallis rank test were applied. A significance level of 5% was used. All collected data are freely available for access and consultation on Zenodo^10^.

**Ethical considerations **

 The study adheres to the bioethical principles proposed by Ezekiel J. Emanuel^11^, including social value, scientific validity, fair participant selection, a favorable risk-benefit ratio, independent review, informed consent, and respect for participants. Regarding the independent review, the research was approved by the Scientific Ethics Committee of the Aysén Health Service under Ordinance N°16 of 2022. Confidentiality of personal identification data was maintained throughout the process by assigning each participant a code.

## Results

The study's results are presented sequentially. First, sociodemographic characteristics are shown, followed by the perceptions of hospitalized patients in relation to the humanized care provided by nurses, and finally, the association of the variables. 

**Sociodemographic characteristics**

The sociodemographic results revealed a rather heterogeneous distribution of patients across hospitalization services. Of the total, 57.38% were admitted to the Surgery Service, 27.87% to the Medicine Service, and 14.75% to the Intermediate Treatment Unit (ITU). Regarding place of origin, 75.41% of participants were from urban areas. There was a slight predominance of women, accounting for 52.46% of the participants. 

More than half of the patients reported being parents. In terms of marital status, 59.02% were single, and 26.23% were married. Additionally, 54.10% reported professing the Catholic religion. Regarding support networks, 98.36% indicated their support came from family members. 

With respect to ethnicity, 31.15% of participants said they identified with the Mapuche ethnic group. Regarding educational level, 80.32% reported having completed basic or intermediate education. In terms of health insurance, 91.80% of the patients were covered by the National Health Fund (FONASA for its Spanish acronym). 

Participant ages ranged from 18 to 84 years, with a mean age of 50 ± 15.83 years. 

**Perception of Behavior Related to Humanized Nursing Care (PCHE)**

The overall results of the application of the instrument showed that 70.49% of the patients perceived humanized care as "always" present, 14.75% as "almost always" present, 9.84% as "sometimes" present, and 4.92% as "never" present. 

**Perception of Behavior Related to Humanized Nursing Care instrument and its dimensions**

The PCHE instrument yielded a favorable overall score, with a mean of 3.74, indicating that participants perceived they "almost always" received humanized nursing care. When analyzed by dimensions, the highest-rated dimension was "quality of nursing," with a minimum score of 2.6, a maximum score of 4.0, and a mean of 3.84. This was followed by "willingness to pay attention," which had a minimum score of 2.0, a maximum of 4.0, and a mean of 3.75. Lastly, the dimension "openness to nurse-patient communication" showed a minimum of 2.3 and a maximum of 4.0, with the lowest mean score of 3.65 (Table 1). 

**Table 1:** Summary of PCHE overall and dimension scores — Hospital in the Southern Zone of Chile, 2023

	Min	x̄ ± SD	Max	P50
PCHE overall score	2.2	3.74 ± 0.36	4.0	3.9
Dimensions				
Quality of nursing	2.6	3.84 ± 0.29	4.0	4.0
Openness to nurse-patient communication	2.3	3.65 ± 0.45	4.0	3.9
Willingness to pay attention	2.0	3.75 ± 0.39	4.0	3.9

Min: Minimum; x̄ ± SD: Mean and Standard Deviation; Max: maximum; P50: 50th percentile

**Association between PCHE score and sociodemographic variables**

A comparison between the PCHE scores and the sociodemographic variables showed no significant association between the variables under study and the PCHE score. Responses were predominantly concentrated in the categories "always" and "almost always" (Table 2). 

**Table 2:** Comparison of the PCHE score by sociodemographic variables — Hospital in the Southern Zone of Chile, 2023

PCHE score					
Variables	Always	Almost always	Sometimes	Never	p
	% (n)	% (n)	% (n)	% (n)	
Sex					0.373
Male	79.31 (23)	13.79 (4)	6.90 (2)	0.00 (0)	
Female	62.50 (20)	15.62 (5)	12.50 (4)	9.38 (3)	
Place of origin					0.695
Urban	71.74 (33)	13.04 (6)	8.70 (4)	6.52 (3)	
Rural	66.67 (10)	20.00 (3)	13.33 (2)	0.00 (0)	
Health insurance					1
FONASA	39 (69.64)	14.29 (8)	10.71 (6)	5.36 (3)	
Non-FONASA	80.00 (4)	20.00 (1)	0.00 (0)	0.00 (0)	
Ethnicity					0.693
Mapuche	63.16 (12)	15.79 (3)	15.79 (3)	5.26 (1)	
Non-mapuche	73.81 (31)	14.29 (6)	7.14 (3)	4.76 (2)	
Education level					0.144
Primary	84.00 (21)	12.00 (3)	0.00 (0)	4.00 (1)	
Secondary	58.33 (14)	12.50 (3)	20.83 (5)	8.33 (2)	
University	66.67 (8)	25.00 (3)	8.33 (1)	0.00 (0)	
Religion					0.784
Catholic	24.00 (24)	6.00 (6)	2.00 (2)	1.00 (1)	
Other religion	11.00 (11)	2.00 (2)	3.00 (3)	1.00 (1)	
None	72.73 (8)	9.09 (1)	9.09 (1)	9.09 (1)	
Marital status					0.364
Single	69.44 (25)	16.67 (6)	11.11 (4)	2.78 (1)	
Married	75.00 (12)	18.75 (3)	0.00 (0)	6.25 (1)	
Widowed	80.00 (4)	0.00 (0)	20.00 (1)	0.00 (0)	
Other	50.00 (2)	0.00 (0)	25.00 (1)	25.00 (1)	
Support network					0.804
Family members	73.81 (31)	11.90 (5)	9.52 (4)	4.76 (2)	
No family members	63.16 (12)	21.05 (4)	10.53 (2)	5.26 (1)	
Hospitalization service					0.117
ITU	100.00 (9)	0.00 (0)	0.00 (0)	0.00 (0)	
Medicine	52.94 (9)	11.76 (2)	23.53 (4)	11.76 (2)	
Surgery	71.43 (25)	20.00 (7)	5.71 (2)	2.86 (1)	
Role in the family group					0.071
Parent	75.00 (30)	5.00 (2)	12.50 (5)	7.50 (3)	
Child	75.00 (6)	25.00 (2)	0.00 (0)	0.00 (0)	
Other	53.85 (7)	38.46 (5)	7.69 (1)	0.00 (0)	

Fisher exact test. %: percentage; n: frequency; ITU: Intermediate Treatment Unit; FONASA: Fondo Nacional de Salud (National Health Fund).

When comparing mean PCHE scores across variables such as sex, place of origin, type of health insurance, ethnicity, education level, religion, marital status, support network, and role within the family group, no statistically significant associations were observed, with responses generally tended toward the categories "always" and "almost always." A significant association was found, however, with the hospitalization service. Specifically, significant differences emerged between the ITU and the Medicine Service, as well as between the ITU and the Surgery Service. The highest mean scores were observed in the ITU, while the lowest scores were found in the Medicine Service, with a tendency toward "always" and "almost always" responses (Table 3). 

**Table 3:** Comparison of the mean PCHE scores by sociodemographic variables — Hospital in the Southern Zone of Chile, 2023

Variables	n	x̄±SD	P50	p
Sex				0.0609*
Male	29	3.8 ± 0.23	3.9	
Female	32	3.7 ± 0.44	3.8	
Place of origin				0.9987**
Urban	46	3.7 ± 0.38	3.9	
Rural	15	3.7 ± 0.29	3.8	
Health insurance				0.5418**
FONASA	56	3.7 ± 0.37	3.9	
Non-FONASA	05	3.8 ± 0.21	3.9	
Ethnicity				0.7410**
Mapuche	19	3.7 ± 0.38	3.9	
Non-mapuche	42	3.8 ± 0.36	3.9	
Education level				0.1701**
Primary	25	3.8 ± 0.37	3.9	
Secondary	24	3.6 ± 0.39	3.8	
University	12	3.8 ± 0.24	3.8	
Religion				0.7406**
Catholic	33	3.8 ± 0.35	3.9	
Other religion	17	3.7 ± 0.33	3.9	
None	11	3.7 ± 0.45	3.8	
Marital status				0.8532**
Single	36	3.8 ± 0.33	3.9	
Married	16	3.7 ± 0.44	3.8	
Widowed	05	3.8 ± 0.26	3.9	
Other	04	3.6 ± 0.50	3.7	
Support network				0.9627**
Family members	42	3.7 ± 0.39	3.9	
No family members	19	3.7 ± 0.30	3.8	
Hospitalization service				**0.0011¥**
ITU	09	4.0 ± 0.07	4.0(a)	
Medicine	17	3.6 ± 0.48	3.8(ab)	
Surgery	35	3.8 ± 0.30	3.8(ac)	
Role in the family group				0.2359¥
Parent	40	3.7 ± 0.42	3.9	
Child	08	3.9 ± 0.19	3.9	
Other	13	3.7 ± 0.25	3.8	

: Frequency; x̄ ± SD: Mean and Standard Deviation; P50: 50th percentile. * T-test for unequal variances; ** Analysis of variance (ANOVA); ¥ Kruskal-Wallis rank test

When comparing the mean PCHE scores in the "qualities of nursing" dimension across sociodemographic variables, no statistically significant associations were observed. Responses showed a tendency toward the "always" category (Table 4). 

**Table 4:** Comparison of the mean PCHE scores in the "qualities of nursing" dimension by sociodemographic variables — Hospital in the Southern Zone of Chile, 2023

Variables	n	x̄±SD	P50	p
Sex				0.2985*
Male	29	3.9 ± 0.22	4.0	
Female	32	3.8 ± 0.35	4.0	
Place of origin				0.9758**
Urban	46	3.8 ± 0.30	4.0	
Rural	15	3.8 ± 0.29	4.0	
Health insurance				0.8692**
FONASA	56	3.8 ± 0.30	4.0	
Non-FONASA	05	3.9 ± 0.20	4.0	
Ethnicity				0.7713**
Mapuche	19	3.8 ± 0.30	4.0	
Non-mapuche	42	3.8 ± 0.30	4.0	
Education level				0.4932**
Primary	25	3.9 ± 0.33	4.0	
Secondary	24	3.8 ± 0.28	3.9	
University	12	3.9 ± 0.25	4.0	
Religion				0.5641**
Catholic	33	3.8 ± 0.32	4.0	
Other religion	17	3.9 ± 0.21	4.0	
None	11	3.8 ± 0.33	3.9	
Marital status				0.9087**
Single	36	3.8 ± 0.27	4.0	
Married	16	3.8 ± 0.40	4.0	
Widowed	05	3.9 ± 0.13	4.0	
Other	04	3.8 ± 0.21	3.9	
Support network				0.5245**
Family members	42	3.8 ± 0.32	4.0	
No family members	19	3.9 ± 0.22	4.0	
Hospitalization service				**0.0796¥**
ITU	09	4.0 ± 0.05	4.0	
Medicine	17	3.7 ± 0.40	3.9	
Surgery	35	3.8 ± 0.26	4.0	
Role in the family group				0.9612**
Parent	40	3.8 ± 0.33	4.0	
Child	8	3.9 ± 0.19	3.9	
Other	13	3.8 ± 0.25	4.0	

n: Frequency; x̄ ± SD: Mean and Standard Deviation; P50: 50th percentile. * T-test for unequal variances; ** Analysis of variance (ANOVA); ¥ Kruskal-Wallis rank test.

A significant association was observed when comparing the mean PCHE scores in the "openness to nurse–patient communication" dimension across hospitalization services, specifically between the ITU and the Medicine Service. In all comparisons, responses tended toward an average rating of "always" (Table 5). 

**Table 5:** Comparison of mean PCHE scores in the "openness to nurse-patient communication" dimension by sociodemographic variables — Hospital in the Southern Zone of Chile, 2023

Variables	n	x̄±SD	P50	p
Sex				0.1030*
Male	29	3.7 ± 0.36	3.9	
Female	32	3.6 ± 0.51	3.8	
Place of origin				0.9545**
Urban	46	3.6 ± 0.49	3.9	
Rural	15	3.6 ± 0.33	3.8	
Health insurance				0.4377**
FONASA	56	3.6 ± 0.47	3.9	
Non-FONASA	5	3.8 ± 0.26	3.9	
Ethnicity				0.9656**
Mapuche	19	3.7 ± 0.47	3.9	
Non-mapuche	42	3.6 ± 0.45	3.8	
Education level				0.3415**
Primary	25	3.7 ± 0.42	4.0	
Secondary	24	3.5 ± 0.52	3.8	
University	12	3.7 ± 0.36	3.9	
Religion				0.4539**
Catholic	33	3.7 ± 0.40	3.9	
Other religion	17	3.5 ± 0.50	3.8	
None	11	3.6 ± 0.55	3.9	
Marital status				0.8978**
Single	36	3.6 ± 0.45	3.8	
Married	16	3.6 ± 0.48	3.8	
Widowed	5	3.8 ± 0.33	4.0	
Other	4	3.6 ± 0.67	3.9	
Support network				0.6749**
Family members	42	3.6 ± 0.47	3.8	
No family members	19	3.7 ± 0.42	3.9	
Hospitalization service				0.0402**
ITU	9	3.9a ± 0.25	4.0	
Medicine	17	3.5ab ± 0.50	3.5	
Surgery	35	3.7c ± 0.44	3.9	
Role in the family group				0.4197**
Parent	40	3.7 ± 0.48	3.9	
Child	8	3.8 ± 0.31	4.0	
Other	13	3.5 ± 0.45	3.8	

n: Fn: Frequency; x̄ ± SD: Mean and Standard Deviation; P50: 50th percentile. * T-test for unequal variances; ** Analysis of variance (ANOVA); ¥ Kruskal-Wallis rank test.

When comparing the mean PCHE scores in the "willingness to care" dimension across sociodemographic variables, no significant associations were observed for place of origin, health insurance, ethnicity, religion, marital status, support networks, or role within the family group. However, significant differences were found based on sex, with men reporting higher average scores. A significant difference was also identified in relation to education level, specifically between participants with primary and secondary education, with those having secondary education showing higher scores. Regarding the hospitalization service, significant associations were found between ITU and Medicine Service and between ITU and Surgery Service, with ITU showing the highest mean score. Across all comparisons, the tendency was for participants to perceive that the willingness to care was "always" present (Table 6). 

**Table 6:** Comparison of mean PCHE scores in the "willingness to care" dimension by sociodemographic variables — Hospital in the Southern Zone of Chile, 2023

Variables	n	x̄±SD	P50	p
Sex				0.0388*
Male	29	3.9 ± 0.20	3.9	
Female	32	3.7 ± 0.48	3.8	
Place of origin				0.9849**
Urban	46	3.7 ± 0.41	3.9	
Rural	15	3.7 ± 0.30	3.8	
Health insurance				0.5528**
FONASA	56	3.7 ± 0.40	3.9	
Non-FONASA	05	3.8 ± 0.19	3.9	
Ethnicity				0.6073**
Mapuche	19	3.7 ± 0.41	3.9	
Non-mapuche	42	3.8 ± 0.38	3.9	
Education level				**0.0102¥**
Primary	25	3.8 ± 0.40	4.0 (a)	
Secondary	24	3.6 ± 0.42	3.8 (ab)	
University	12	3.8 ± 0.21	3.8 (c)	
Religion				0.7347**
Catholic	33	3.8 ± 0.38	3.9	
Other religion	17	3.8 ± 0.34	3.9	
None	11	3.7 ± 0.48	3.8	
Marital status				0.6358**
Single	36	3.8 ± 0.33	3.9	
Married	16	3.8 ± 0.49	3.9	
Widowed	05	3.8 ± 0.29	3.8	
Other	04	3.5 ± 0.59	3.6	
Support network				0.7280**
Family members	42	3.8 ± 0.42	3.9	
No family members	19	3.7 ± 0.32	3.8	
Hospitalization service				**0.0005¥**
ITU	09	4.0 ± 0.00	4.0 (a)	
Medicine	17	3.5 ± 0.55	3.8 (ab)	
Surgery	35	3.8 ± 0.29	3.9 (ac)	
Role in the family group				0.2286¥
Parent	40	3.7 ± 0.45	3.9	
Child	08	3.9 ± 0.17	3.9	
Other	13	3.7 ± 0.22	3.8	

n: Fn: Frequency; x̄ ± SD: Mean and Standard Deviation; P50: 50th percentile. * T-test for unequal variances; ** Analysis of variance (ANOVA) and Sidak post hoc comparisons; ¥ Kruskal-Wallis rank test.

## Discussion

The study found that adult hospitalized patients perceived humanized nursing care favorably. When instrument responses were categorized according to the Likert scale, the most frequent response was "always," accounting for 70.49% of the total. However, this result is lower when compared to a Chilean study conducted in the Hemato-Oncology Unit that reported a higher score of 86%^12^. In contrast, a study conducted in Mexico reported lower scores, with 66.7% of participants selecting "almost always" and only 16.1% selecting "always."^13^. 

Regarding the dimensions of the PCHE instrument, the highest rated was "qualities of nursing," with a mean score of 3.84, indicating that patients' perceptions fell between "almost always" and "always." When comparing the items of this dimension, the highest scores were observed in items 7 and 17, with 91.80% and 90.16% of participants, respectively, stating that they felt calm when accompanied by a nurse and that their beliefs and values were respected during hospitalization. These results exceeded those reported in the study by Monje et al.^4^ and align with the Code of Ethics of the Chilean College of Nurses, which establishes guidelines regarding dignified care and the nurse’s obligation to respect patients' values, customs, and spiritual beliefs. Similar results are found in a study conducted in Peru^14^. In contrast, the lowest score was recorded for item 1, where only 8.20% of participants felt that nursing staff consistently made them feel like a person. 

The second-highest rated dimension was "willingness to pay attention," with a mean score of 3.75. The highest-rated item was item 16, with 95.08% of participants indicating that nurses always addressed them by name. In contrast, the lowest score was recorded for item 22, with 63.93% of patients reporting that nurses do not always respond promptly to their calls during hospitalization. This last point is particularly relevant, as a timely response is considered a key attribute of quality in healthcare. These results contrast with those of a study conducted in Argentina, where this dimension was the highest rated by patients, who considered that nurses had a quality interpersonal relationship with the person in their day-to-day care^15^. 

The lowest-rated dimension was "openness to nurse-patient communication," with a mean score of 3.65. The highest scores were observed in items 5 and 11, both at 81.97%. In these items, patients reported that nurses took the time to clarify their concerns and responded clearly and confidently to their questions. In contrast, the lowest score was recorded for item 12, at 65.57%, where patients indicated that nurses did not always introduce themselves by name and role before performing procedures. This finding is consistent with a study conducted in a Hemato-Oncology Unit, where this item also received a relatively low score (76.5%)^12^. 

When average PCHE scores were compared with sociodemographic variables (sex, place of origin, health insurance, ethnicity, education level, religion, marital status, support network, and role within the family group), no statistically significant associations were observed. The only significant association emerged in relation to the hospitalization service, where the ITU received the highest average scores and the Medicine Service the lowest, with responses tending toward the "always" and "almost always" categories. This result may be related to the number of patients cared for in each service. However, a study conducted in Argentina reported significant differences based on patients' education levels: individuals with lower levels of education (no education, primary, or secondary education) had a more positive perception of humanized care^15^.

Finally, the mean scores for the "willingness to care" dimension of the PCHE instrument were compared across sociodemographic variables. No significant associations were observed, except for sex (with men reporting higher average scores) and educational level, where a significant difference was observed between participants with primary and secondary education, the latter showing higher scores. In relation to the hospitalization service, the highest ratings were found in the ITU. These results contrast with those of a study conducted in a Hemato-Oncology Unit, which reported a significant association with age, with lower scores among participants aged 18 to 49^12^. 

## Conclusions

The study determined that patients in a public hospital perceived nursing care as humanized and established that there was no significant association between sociodemographic variables and the score of the third version of the PCHE instrument. 

When it was analyzed by dimensions, the average scores for each dimension and each individual item were calculated, which made it possible to provide feedback to areas with the lowest scores and to reinforce those that received higher scores. It is important to remember that the art of caring is a dynamic, open, and continuous process. Therefore, ongoing feedback is essential to enhance the quality of care. 

Given that the lowest average score was recorded in the "openness to nurse-patient communication," dimension—focused on active listening, dialogue, presence, and understanding of the person receiving care—it is essential to create strategies that help strengthen communication in care. Communication is not just a process but rather an important resource that enables nurses to establish transpersonal relationships with their patients^16^. Equally important is the cultivation of observation skills among nursing professionals, as this enables them to recognize human beings in their essence and make informed care decisions. In addition, it will allow them to search for specific care rather than relying solely on standard routines. On the other hand, active listening must be fostered in order to discern what is most meaningful to the person. This requires nurturing a sensitive spirit capable of grasping the whole human essence. Such an approach fosters compassion and empathy to navigate complex situations with understanding—always grounded in respect for human dignity^17^. 

Several authors who reflect on care issues have expressed that “a work may be technically perfect and still lack art; therefore, it is essential to unite elements such as technique, soul, and mind.”^17^ For this reason, it is important to continue strengthening these lines of health research and promote more respectful care that ensures not only sound treatments but truly humanized care within hospital settings. Likewise, evaluating the creation of an institutional protocol for humanized care is also important. 

One of the limitations of this study was the sample size, which was influenced by the specific characteristics of the hospitalized patients in the services. 
